# Combining Radiotherapy and Immunotherapy in Metastatic Breast Cancer: Current Status and Future Directions

**DOI:** 10.3390/biomedicines10040821

**Published:** 2022-03-31

**Authors:** Steven David, Jennifer Tan, Shankar Siva, Lama Karroum, Peter Savas, Sherene Loi

**Affiliations:** 1 Department of Radiation Oncology, Peter MacCallum Cancer Centre, Melbourne, VIC 3000, Australia; jennifer.tan@petermac.org (J.T.); shankar.siva@petermac.org (S.S.); lama.karroum@petermac.org (L.K.); 2 The Sir Peter MacCallum Department of Oncology, University of Melbourne, Melbourne, VIC 3000, Australia; peter.savas@petermac.org (P.S.); sherene.loi@petermac.org (S.L.); 3 Department of Cancer Medicine, Peter MacCallum Cancer Centre, Melbourne, VIC 3000, Australia

**Keywords:** SABR, breast cancer, immunotherapy, stereotactic radiotherapy, immuno-oncology

## Abstract

The role of radiotherapy and immunotherapy with immune checkpoint inhibitors (ICI) is of emerging interest in many solid tumours, including breast cancer. There is increasing evidence that the host’s immune system plays an important role in influencing the response to treatment and prognosis in breast cancer. Several pre-clinical studies and clinical trials have reported on the ‘abscopal effect—regression of distant untreated tumour sites, mediated by an immunological response following ionizing radiation to a targeted tumour site. Stereotactic Ablative Body Radiotherapy (SABR) is a non-invasive technique used to augment various immune responses with an ablative tumoricidal dose when compared to conventional radiotherapy. SABR is characterized by typically 1–5 precision radiotherapy treatments that simultaneously deliver a high dose, whilst sparing normal tissues. Following SABR, there is evidence of systemic immune activation in patients with increased PD1 expression on CD8^+^ and CD4^+^ T cells. Studies continue to focus on metastatic triple-negative disease, a highly immunogenic subtype of breast cancer with poor prognosis. In this review, we discuss the immunological effect of SABR, alone and in combination with immunotherapy, and the importance of dose and fractionation. We also propose future strategies for treating oligometastatic disease, where this approach may be most useful for producing durable responses.

## 1. Introduction

Immuno-oncology (I-O) is now a well-established pillar of cancer therapy that focuses on engaging the host’s immune system to eradicate tumours indirectly. It is well known that the host immune system plays a role in responses to traditional breast cancer treatments, as evidenced by the presence of higher levels of tumour-infiltrating lymphocytes (TILs), predicting for better survival and response to neoadjuvant chemotherapy, in early-stage triple-negative breast cancer [1]. Immunotherapeutic agents directly engage with the host immune system, aiming to produce durable responses due to the memory of the host’s adaptive immune system towards tumour-specific antigens, which may provide ongoing and long-term tumour control, even when therapy is ceased. This reflects, in part, the highly efficacious nature of immunotherapy in some cancers, apparently exterminating all viable malignancy in a small proportion of patients with otherwise incurable disseminated disease [2]. For most patients where cure is not achieved, IO can, nevertheless, generate persistent anti-tumour immune responses, through favourable manipulation of the tumour–immune microenvironment, which provides long-term tumour control in the absence of continual therapy.

Scientists have explored several I-O agents to engage and enhance the host’s immune system. The programmed cell death protein 1 (PD-1)/programmed death-ligand 1 (PD-L1) pathway is the most widely studied in breast cancer. This is an immune checkpoint pathway that acts as a handbrake on a normal functioning immune system to prevent excessive immune responses to frequently encountered pathogens, and also to suppress autoimmunity [3,4]. Tumours employ a variety of mechanisms to escape immunosurveillance. One of these mechanisms is the up-regulation of inhibitory immune checkpoints, in particular, the PD-1/PDL1 pathway [5].

Scientists have developed monoclonal antibodies focused on immune checkpoints, including PD-1 and PD-L1, to re-engage the host’s immune system. This class of drugs, termed immune checkpoint inhibitors (ICIs), has been successful in many malignancies with other checkpoint targets. Ipilimumab, for use in advanced melanoma, was the first approved ICI in 2011, and is a drug that targets the cytotoxic T-lymphocyte-associated protein 4 (CTLA-4) checkpoint. Since that time, anti-PD-1/PD-L1 antibodies have shown broad efficacy, in several otherwise difficult-to-treat advanced cancers, and constitute a *bona fide* revolution in cancer therapy. More recently, the efficacy of these agents has been demonstrated with the neoadjuvant and adjuvant setting, for several tumour types.

The success of immunotherapy in other tumour types has resulted in continued research efforts, focusing on their potential role in breast cancer. This potential would be most beneficial in the advanced metastatic triple-negative breast cancer setting due to poor prognosis and a pressing need to improve treatment outcomes.

Radiotherapy has a direct cytotoxic effect on tumour cells, inducing DNA damage. There is an increasing body of pre-clinical and clinical evidence that radiotherapy can induce immunological effects, which can be combined with immunotherapy to potentially produce and enhance systemic immune response. In this review, we discuss (1) the current clinical status of immunotherapy in breast cancer, (2) the immunological effects of radiotherapy, (3) the potential role of dose and fractionation in immune potentiation, (4) evidence supporting the optimal sequencing of radiotherapy and immunotherapy, (5) pre-clinical evidence of the immune potentiation effects of stereotactic ablative body radiotherapy (SABR), (6) clinical evidence of the immunological effects of SABR, (7) targeting of oligometastatic disease, (8) combination studies of immunotherapy and radiotherapy in breast cancer, and (9) future directions.

## 2. Immunotherapy in Breast Cancer—Current Clinical Status

Breast cancer is the second most common malignancy diagnosed worldwide [6]. Approximately 30% of patients will ultimately develop metastatic disease [7]. Triple-negative breast cancer (TNBC) represents approximately 15% of all breast cancer, with the poorest prognosis [8,9,10] and least effective treatment options. TNBC is characterised by a high mutational burden, together with relatively high levels of PD-L1 expression [11], making it an ideal breast cancer phenotype to research the efficacy of ICIs.

Initial early phase trials investigating monotherapy with ICIs were underwhelming. Atezolizumab has demonstrated modest efficacy and acceptable tolerability in a phase IA trial of participants with metastatic TNBC, in which 85% of participants received ≥ 4 prior systemic regimens. The unconfirmed RECIST ORR was 24% and the 24-wk PFS rate was 33% [12,13]. The use of Pembrolizumab for participants selected for PD-L1-positive expression, resulted in objective response rates around 20% in the first line setting and 5% in the second line and beyond, with no PD-L1 selection. This data clearly implied that earlier line treatment in advanced TNBC participants is prudent [14,15].

Given the modest responses seen with PD-1/PD-L1 monotherapy, efforts have been made to evaluate treatment in combination with existing and novel therapies to explore synergies with immunotherapy and increase response rates. A phase IB trial [16] hypothesized that combining atezolizumab with nab-paclitaxel chemotherapy in metastatic TNBC would enhance tumour-specific T-cell immunity, by exposing the immune system to high levels of tumour antigens and modulating T-cell and NK cell functions [16]. The combination arm of this trial demonstrated an ORR of 70.8% in participants who had received no prior therapy in the advanced setting, with no new safety signals. This response was independent of PD-L1 status.

The IMpassion130 trial demonstrated that among patients with untreated metastatic triple-negative breast cancer with PD-L1–positive tumours, treated with nab-paclitaxel and atezolizumab or placebo, the median overall survival was 25.0 months and 15.5 months, respectively (hazard ratio, 0.62; 95% CI, 0.45 to 0.86) [17], resulting in FDA accelerated approval. There was no appreciable benefit in the PD-L1-negative patients. Patients were required to be 12 months or more from last adjuvant or neoadjuvant chemotherapy dosing and many patients were de novo metastatic. Recently, results from the KEYNOTE-355 phase III study that explored pembrolizumab with a variety of chemotherapy regimens, in patients that were ≥ 6 months from last chemotherapy regimen, were reported [18]. A significant and clinically meaningful benefit was seen in patients that were PD-L1 positive, defined using the Merck Dako assay, as a Combined Positive Score (CPS) of ≥10 with regards to improved progression-free survival, resulting in the FDA granting priority review for accelerated approval. In a subsequent report, this benefit in patients with tumours, showing CPS ≥ 10, was also seen in overall survival: 23 months with pembrolizumab/chemotherapy versus 16.1 months with chemotherapy alone (hazard ratio 0.73; 95% CI, 0.55 to 0.95) [19]. Although anti-PD-1/PD-L1 therapy in combination with chemotherapy is a promising option for PD-L1-positive disease, these results also highlight the lack of effective therapies for the many patients who are designated PD-L1 negative and the need for new strategies to achieve deeper and more durable responses for PD-L1-positive patients.

There are minimal data supporting the efficacy of immunotherapy in other breast cancer phenotypes. In unselected ER+Her2- breast cancers, results to date have been underwhelming [20,21,22]. In HER2+ breast cancer, results have been more promising, but remain modest in early phase clinical trials [23,24].

## 3. Immunological Effects of Radiotherapy

**Figure 1:**
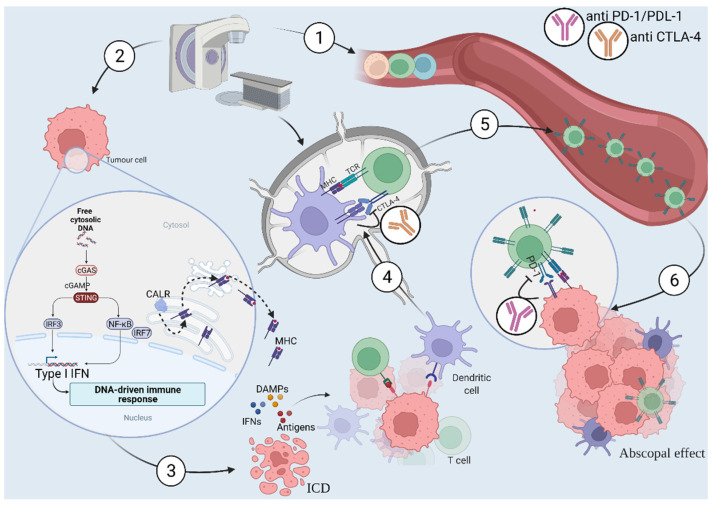
(1) Radiotherapy leads to tissue damage, including immune cell depletion. (2) Radiotherapy has a direct cytotoxic effect on tumour cells inducing DNA damage leading to activation of the cGAS/STING pathway resulting in: (3) immunological cell death (ICD) and activation of inflammatory signals including the release of tumour-associated antigenic peptides (antigens), damage-associated molecular patterns (DAMPs), calreticulin (CALR) expression and major histocompatibility class I (MHC I) surface expression. This up-regulation leads to activation of dendritic cells (DCs) that (4) migrate to lymph nodes resulting in antigen presentation and subsequent (5) priming of tumour-specific T cell and proliferation. (6) Primed T-cells then attack tumours located within the irradiated field and in distant locations (abscopal effect). This response can be enhanced by addition of immune checkpoint inhibitors (ICIs) such as anti PD-1/PDL-1 and anti CTLA-4 agents to counteract the immunosuppressive effect of Radiotherapy.

Radiotherapy is known for its direct cytotoxic effects on tumour cells, inducing DNA damage. There is an increasing body of pre-clinical and clinical evidence supporting the concept that radiotherapy can have immunological effects, resulting from an immunological cell death (ICD) [25,26]. Treating a single tumour with radiation can result in an immune response against tumours elsewhere in the body. This is known as the abscopal effect. Several key mechanisms are involved. These mechanisms include the generation of tumour-associated antigenic peptides through cell death and the release of “danger signals”, including major histocompatibility class I surface expression, which is up-regulated in a dose dependent fashion, via mTOR activation to subsequently present tumour antigens to the cell surface for recognition by CD8-positive T cells [27]; calreticulin expression, which promotes phagocytosis [28]; and the release of high-motility group box 1 (which stimulates the immune response via toll-like receptor 4 [29]). This leads to activation of dendritic cells (DCs) that migrate to lymph nodes, resulting in antigen presentation and subsequent tumour-specific T cell activation and proliferation. In addition to this process is the DNA damage caused by radiation, resulting in the release of DNA fragments that are transferred from the nucleus to the cytoplasm. This transfer activates the cGAS/STING pathway, produces type I interferon, cytokines and chemokines that enable recruitment of immune cells to the tumour microenvironment and cross-priming of DCs [30,31,32]. This process augments immune responses, increases vascular permeability and promotes homing of a tumour-directed immune response [33,34]. Conversely, some effects of radiation may be detrimental to the immune response, including the recruitment of regulatory T cells, inhibitory macrophages, other myeloid-derived suppressor cells [35,36,37,38], as well as immunosuppressive cytokines (such as TGF-beta) and chemokines [39]. In addition, radiation-induced up-regulation of PD-L1, a potent inhibitor of immune activation, within the tumour micro-environment has been demonstrated [40]. The use of targeted antibodies to inhibitory targets, such as PD-L1, has been the focus of current research to synergise and complement the immunological effects of radiotherapy. Research is focusing on the optimal combination of radiotherapy (including dose and fractionation regimens) and immunotherapy. Figure 1 schematically illustrates this process, as well as immunotherapy agents currently being studied, which may potentiate the immune response.

## 4. Potential Role of Dose and Fractionation in the Immunological Effects of Radiotherapy

The full immunological potential of radiotherapy may be influenced by the radiation dose and fractionation [41]. SABR is a highly focused, non-invasive, targeted technique that delivers 1–5 high-dose radiation treatments. The primary advantage of SABR over fractionated external beam radiotherapy is its ability to spare surrounding normal tissue, while intensifying the radiation dose to the tumour. This may be particularly beneficial through avoidance of irradiating draining lymph nodes and consequent lymphopenia. Another potential advantage is that the ablative hypofractionation dose spectrum employed by SABR techniques heralds a potential for greater augmentation of the immune response [42]. This is addressed in more detail in Section 6.

## 5. Sequencing of Radiotherapy and Immunotherapy

There is a lack of evidence on the optimal sequencing of radiotherapy and immunotherapy in breast cancer. However, a pre-clinical prostate cancer study of transgenic mice suggested anti-tumour responses were dependent on the timing of RT and immunotherapy. The mice received a single high dose of radiation and an immunogenic vaccine. Maximal tumour responses were observed when immunotherapy was administered 3–5 weeks after radiation, but not if administered either prior to or later than 5 weeks after radiation [43].

Furthermore, in non-small cell and small cell lung cancer, pre-clinical and clinical evidence has demonstrated superior treatment efficacy and improved immune-related progression-free survival when chemotherapy was given prior to immunotherapy, as opposed to concurrently with immunotherapy [44,45].

These studies support the hypothesis that the activation and augmentation of an immune response by immunotherapy may be more effective if prior radiation or chemotherapy administration had generated de novo tumour antigens and created favourable tumour microenvironments for an effective immune response.

However, some pre-clinical evidence suggests that the optimal sequencing is variable and may be dependent on the mechanism of action of the investigational agent. Young et al. administered an anti-CTLA-4 antibody or an anti-OX40 agonist antibody, either before or after 20 Gy (a tumoricidal SABR dose) was delivered to a tumour within a mouse model [46]. Anti-CTLA-4 was most effective when given prior to SABR, mostly due to regulatory T-cell depletion. Conversely, the anti-OX40 agonist was more effective when delivered after, partly due to its effect on increasing the numbers of activated CD8^+^ T cells when delivered one day after radiotherapy. This indicates that the type of the immunotherapy agent selected could influence the optimal scheduling of treatment modalities. Retrospective clinical data has demonstrated that concurrent radiotherapy and ICI may yield superior tumour control and survival compared with sequential therapy [47,48]. Timing, choice of agent and radiation dose and fractionation are all areas requiring further research, to optimise any synergistic benefits between radiotherapy and ICI.

In addition to checkpoint inhibition, dendritic cell expansion in combination with radiotherapy further supports the hypothesis of the immunostimulatory effects of radiotherapy. Initial evidence on the abscopal effect emerged from a study of mice, bearing two sites of syngeneic mammary carcinoma, treated with a single dose of 2 or 6 Gy to one site only, followed by a growth factor (Flt3-Ligand) daily for 10 days. The radiotherapy alone led to a tumour response only in the irradiated site; however, with the addition of sequential Flt3-L, a tumour response was also observed in the non-irradiated site [49].

## 6. Preclinical Evidence of the Immune Potentiation Effects of SABR

Immunogenic responses at sites distant to the SABR-targeted sites have been observed and reported by our institution [50] and in the wider literature [51]. Ablative doses, when compared to conventional doses, result in a greater degree of stromal and vascular damage, ceramide-induced endothelial cell damage and increased apoptosis of tumour cells [52,53]. The tumour microenvironment becomes enriched with tumour-derived antigens, with co-existing dendritic cell (DC) activation, antigen cross-presentation and tumour-specific T cell responses. Lee et al. showed that in a B16 mouse melanoma model, tumour inhibition was more pronounced with ablative doses of radiation, as compared to conventional radiation [54]. A single dose of 15 Gy in the draining lymph nodes has been shown to induce significant cross-priming of T-cells against tumour antigens [55]. Different SABR regimens have been tested in combination with anti-CTLA-4 antibodies (20 Gy in one fraction, 8 Gy in three fractions of 6 Gy in five fractions) in a murine model. Each regimen demonstrated significant tumour growth delay within the irradiated field; however, the abscopal response in tumours outside the field was only observed with the fractionated regimens [56].

In triple-negative breast cancer mouse models, combinations of immunostimulatory antibodies, including anti-PD-1 antibody, enhance the anti-tumour effect of radiotherapy. Verbrugge et al. [57] found PD-1 signalling inhibition was critical to synergistic effects with radiotherapy, to promote rejection of triple-negative breast cancer in mice. They identified that a single dose of 12 Gy did not adversely affect the established proinflammatory immune cells that characterise an effective immune response. These cells include tumour-infiltrating lymphocytes (TILs), such as Type 1 T helper cells, cytotoxic CD8 T cells, natural killer cells, dendritic cells and M1 macrophages [58,59]. Notably, the tumour microenvironments of irradiated mammary tumours were enriched with more functionally active, tumour-specific T cells and Ly-6C^+^ memory CD8^+^ T cells [57].

More recently, Vanpouille-Box et al. unravelled a potential mechanism of the most effective radiotherapy dose scheduling, to induce a systemic proinflammatory tumour microenvironment, while inhibiting immune suppressor TILs [31]. Their experiments found single doses of 12–18 Gy or higher would generate DNA exonuclease Trex1 that prohibits an effective immune response. However, a fractionated regimen of 3 × 8 Gy not only prevented Trex1 induction, but amplified interferon-beta production, essential for mediating an abscopal effect via downstream effects on priming CD8^+^ T cells. These differences in dose/fractionation have yet to be replicated in a clinical setting and future studies are required to determine the optimal schedule.

## 7. Clinical Evidence of the Immunological Effects of SABR

Despite preclinical evidence supporting the synergistic effects of combining radiotherapy and ICI, clinical evidence has not been forthcoming in breast cancer and other tumour types.

In a recent randomised trial of 62 patients with metastatic head and neck squamous cell carcinoma, nivolumab was administered, with or without SABR [60]. SABR was 3 × 9 Gy delivered to only one metastatic site, with one other untreated site for comparison. It was a negative trial with no difference in objective response rate found in non-irradiated sites. The authors hypothesised that this may, in part, be due to irradiating only a single metastatic site. It has been postulated that treating multiple sites in different locations with different tumour microenvironments may release a broader array of tumour-associated antigens, for deeper, more effective synergistic response with ICI.

A phase II randomised controlled trial in advanced non-small cell lung (NSCLC) cancer (Pembro-RT trial) randomised participants to pembrolizumab alone (control arm) or SABR to one metastasis, followed by pembrolizumab (experimental arm). Whilst there was a doubling of response rates in the SABR arm (36% vs. 18%), it did not meet the study criteria for statistically meaningful benefit [61].

Nevertheless, a pooled analysis of two randomised trials in advanced NSCLC, which compared pembrolizumab to pembrolizumab plus radiotherapy, showed a significant increase in overall response rate (ORR) in non-irradiated lesions of patients who received radiotherapy [62]. There was also a significant improvement in progression-free survival (4.4 months versus 8.3 months—*p* = 0.046) and overall survival (9.2 months versus 19.2 months) in the radiotherapy arm. Interestingly, in the subgroup that received ablative doses of radiotherapy (24 Gy in three fractions or 50 Gy in four fractions), the ORR was significantly higher than in those receiving non-ablative doses of radiotherapy (45 Gy in 15 fractions). This supports our hypothesis that SABR may be more immunostimulatory then conventional radiotherapy.

Our interpretation of the available data is that targeting a single lesion in a widely metastatic patient may not yield the promising results observed in the pre-clinical settings. The genomic heterogeneity of different metastatic sites in metastatic breast cancer [63], is likely to have implications for variable immune-mediated effects of SABR on each metastatic site. Tang et al. observed greater T-cell activation when SABR plus ICIs were delivered to the liver compared to lung metastatic sites, suggesting immunogenicity may also be dependent on the target site for irradiation [64].

## 8. Targeting Oligometastatic Disease

There is emerging evidence on an alternate strategy to targeting a single-site metastasis with radiotherapy. The concept of oligometastatic disease is defined by a state of limited metastatic dissemination, for which local ablative therapy to all sites of visible metastatic disease could be curative.

There are minimal published breast-cancer-specific prospective data assessing SABR in oligometastatic disease; however, several trials are actively recruiting. David et al. [65] recruited 15 patients, with 1–3 bone only metastases, and investigated the feasibility and efficacy of SABR to a dose of 20 Gy, in one fraction to each metastasis. The treatment was well tolerated, with no grade 3 toxicities. Lesion local control was excellent at 100% at 2 years and distant progression-free survival PFS was 67% at 2 years. Trovo et al. [66] reported on a prospective phase II trial of 54 patients with 1–5 oligometastases. Similarly, the treatment was well tolerated, with an impressive 2-year lesion local control and PFS of 97% and 53%, respectively. Lastly, Milano et al. [67] demonstrated impressive longer-term results in 40 patients receiving curative intent SABR to 1–5 oligometastases. At 4 years, lesion local control was 89%, and distant PFS was 38%, with no grade 4 or 5 toxicities observed.

Recently, Palma et al. published a phase II randomised trial in patients with oligometastatic malignancy. Treating all sites of visible metastatic disease (1–5 metastases) compared to standard of care systemic therapy with SABR resulted in an improvement in the 5-year overall survival of 25% (17.7% in the standard of care arm versus 42.3% in the SABR arm, *p* = 0.006) [68]. This study was conducted in the pre-immunotherapy era. The subset of patients with breast cancer was 20% of the patients that received SABR.

These data, taken together, support the hypothesis that SABR to all visible oligometastases may translate into durable systemic disease control.

Several ongoing, large randomised trials will investigate this approach further, in oligometastatic breast cancer (Table 1)

Baseline tumour burden is a prognostic factor for patients with melanoma and non–small-cell lung cancer treated with immunotherapy. Tarantino et al. recently reviewed data on solid organ tumours treated with new generation immuno-oncology agents and found lower baseline tumour burden is associated with better outcomes [75].

Logically, ICIs may be most effective in patients with low disease burden, treated with maximal local radiotherapy [76]. A trial in oligometastatic renal cell carcinoma demonstrated ORR of 63% and median PFS of 15.6 months, with the combination of SABR to all sites of disease and pembrolizumab [77]. These outcomes are approximately double that of results from pembrolizumab monotherapy [78]. In earlier stage disease, the addition of PDL-1 antibody Durvalumab (versus placebo) to radical chemoradiotherapy for Stage III NSCLC, a disease with a high rate of occult metastases, demonstrated an improvement in the median overall survival from 36.3 months to 49.6 months [79].

## 9. Combination Studies of Immunotherapy with Radiotherapy in Breast Cancer

Formenti et al. reported a randomized controlled trial of two doses of fresolimumab (TGF beta-blocking antibody), given in patients with metastatic breast cancer. Targeted radiotherapy 3 × 7.5 Gy was delivered to one metastatic tumour site and 7 weeks later to a second site. They concluded that both treatments were feasible and safe. Patients who received the higher dose of fresolimumab had an improved median survival, which correlated with evidence of systemic immune activation [80]. A small single-arm phase 2 trial enrolled 17 patients with metastatic TNBC. Radiotherapy was administered to a metastatic site to a dose of 30 Gy in five fractions. The treatment was safe with an encouraging activity and an overall response rate (ORR) of 17.6 % [81]. A larger trial of 67 patients in advanced TNBC were treated with nivolumab and either radiotherapy to a metastatic site (3 × 8 Gy), cyclophosphamide, cisplatin or doxorubicin. The most impressive response rates were seen in the cisplatin (ORR of 23%) and doxorubicin (ORR 35%) groups [82].

A recently completed trial recruited 52 patients (AZTEC: NCT03464942) and aims to investigate the most optimal radiotherapy dose and fractionation, in combination with atezolizumab (ICI). It is a phase II randomised trial comparing single 20 Gy to 3 × 24 Gy to 1–4 metastases (with a minimum of one untreated site), followed by atezolizumab. The translational component of this study should provide valuable information on the effects of radiotherapy fractionation schedules on the immune system.

We have now completed our single-arm institutional Phase IB Pilot Study of Stereotactic Ablation for Oligometastatic Breast Neoplasia in combination with Anti-PD-1 Antibody Pembrolizumab (BOSTON II) [83]. Our hypothesis was that stereotactic ablative body radiotherapy (SABR) is more immunogenic than conventional radiation therapy. Early results [84] have demonstrated the feasibility and safety of this combination, with evidence of systemic immune activation in responders compared to non-responders. Responders have increased PD1 expression on CD8^+^ and CD4^+^ T cells. CD8^+^ T_EMRA_ cells and EOMES^+^ Tbet^-^ CD8^+^ T cells were also higher in responders. Excitingly, seven patients with oligometastatic ER+ disease remained with no evaluable disease 2 years from trial enrolment, with six of seven having PD-L1-negative tumours. This, combined with the correlative evidence of peripheral immune activation, suggests that these patients have developed anti-tumour immunity. It is also promising when considering the KEYNOTE 28 study [20] results, which suggested a modest, but durable, response with pembrolizumab monotherapy in previously treated PD-L1-positive, ER+/HER2- BC patients.

## 10. Future Directions

Combining immunotherapy, chemotherapy and radiotherapy approaches is promising, especially where few effective long-term therapeutic options exist in triple-negative breast cancer. The translation of the abscopal effect from pre-clinical studies to clinical reality, even in combination with ICI, has not been successful, raising the question of whether different approaches need to be considered. Our reading of the pre-clinical and clinical evidence presented in this review indicates that future research with the greatest chance of success should focus on patients with oligometastatic breast cancer (either de-novo or induced by systemic therapy) and combining SABR to all metastatic sites, in combination with ICI (in addition to chemotherapy). Reducing the systemic disease burden with cytoreduction, in combination with immunotherapy, could provide the optimal environment to induce a sustained systemic response, with clinically meaningful outcome.

## Figures and Tables

**Table 1:** Ongoing randomised trials investigating SABR in oligometastatic breast cancer.

Trial	Design	Participant Number	Primary Endpoint
Trial of Superiority of Stereotactic Body Radiation Therapy in Patients with Breast Cancer (STEREO-SEIN) * NCT02089100 [69]	Multicentric Phase III Trial	n = 280	PFS
Standard of Care Therapy With or Without Stereotactic Radiosurgery and/or Surgery in Treating Patients With Limited Metastatic Breast Cancer * NCT02364557 [70]	Randomised phase IIR/III Trial	n = 402	PFS and OS
Metastases-directed Radiotherapy in Addition to Standard Systemic Therapy in Patient with Oligometastatic Breast Cancer (OLIGOMA) * [71]	Randomised controlled multi-national, multicentre therapeutic confirmatory trial	n = 564	PFS and quality of life
Standard Treatment ± SBRT in Solid Tumours Patients With Between 1 and 3 Bone-only Metastases (STEREO-OS) [72]	Randomised, Phase III trial	n = 196	PFS
Conventional Care Versus Radioablation (Stereotactic Body Radiotherapy) for Extracranial Oligometastases (CORE) [73]	Multi-centre phase II/III randomised controlled trial	n = 245	PFS
A Randomized Phase III Trial of Stereotactic Ablative Radiotherapy for the Comprehensive Treatment of 4–10 Oligometastatic Tumours (SABR-COMET 10) [74]	Randomised Phase III study	n = 159	OS

* Trials specifically recruiting breast cancer patients.

## Data Availability

Not applicable.

## References

[1] Loi S., Drubay D., Adams S., Pruneri G., Francis P.A., Lacroix-Triki M., Joensuu H., Dieci M.V., Badve S., Demaria S. (2019). Tumor-Infiltrating Lymphocytes and Prognosis: A Pooled Individual Patient Analysis of Early-Stage Triple-Negative Breast Cancers. J. Clin. Oncol..

[2] Larkin J., Chiarion-Sileni V., Gonzalez R., Grob J.-J., Rutkowski P., Lao C.D., Cowey C.L., Schadendorf D., Wagstaff J., Dummer R. (2019). Five-Year Survival with Combined Nivolumab and Ipilimumab in Advanced Melanoma. N. Engl. J. Med..

[3] Prokunina L., Castillejo-López C., Öberg F., Gunnarsson I., Berg L., Magnusson V., Brookes A.J., Tentler D., Kristjansdóttir H., Gröndal G. (2002). A regulatory polymorphism in PDCD1 is associated with susceptibility to systemic lupus erythematosus in humans. Nat. Genet..

[4] Nishimura H., Okazaki T., Tanaka Y., Nakatani K., Hara M., Matsumori A., Sasayama S., Mizoguchi A., Hiai H., Minato N. (2001). Autoimmune Dilated Cardiomyopathy in PD-1 Receptor-Deficient Mice. Science.

[5] Wei S.C., Duffy C.R., Allison J.P. (2018). Fundamental Mechanisms of Immune Checkpoint Blockade Therapy. Cancer Discov..

[6] Siegel R.L., Miller K.D., Goding Sauer A., Fedewa S.A., Butterly L.F., Anderson J.C., Cercek A., Smith R.A., Jemal A. (2020). Cancer statistics, 2020. CA Cancer J. Clin..

[7] Redig A.J., McAllister S.S. (2013). Breast cancer as a systemic disease: A view of metastasis. J. Intern. Med..

[8] Banerjee S., Reis-Filho J.S., Ashley S., Steele D., Ashworth A., Lakhani S., E Smith I. (2006). Basal-like breast carcinomas: Clinical outcome and response to chemotherapy. J. Clin. Pathol..

[9] Bauer K.R., Brown M., Cress R.D., Parise C.A., Caggiano V. (2007). Descriptive analysis of estrogen receptor (ER)-negative, progesterone receptor (PR)-negative, and HER2-negative invasive breast cancer, the so-called triple-negative phenotype: A population-based study from the California cancer Registry. Cancer.

[10] Bianchini G., Balko J.M., Mayer I.A., Sanders M.E., Gianni L. (2016). Triple-negative breast cancer: Challenges and opportunities of a heterogeneous disease. Nat. Rev. Clin. Oncol..

[11] Mittendorf E.A., Philips A.V., Meric-Bernstam F., Qiao N., Wu Y., Harrington S., Su X., Wang Y., Gonzalez-Angulo A.M., Akcakanat A. (2014). PD-L1 Expression in Triple-Negative Breast Cancer. Cancer Immunol. Res..

[12] Emens L.A., Braiteh F.S., Cassier P.A., Delord J.-P., Eder J.P., Fasso M., Xiao Y., Wang Y., Molinero L., Chen D.S. (2015). Inhibition of PD-L1 by MPDL3280A leads to clinical activity in patients with metastatic triple-negative breast cancer (TNBC). Cancer Res..

[13] Nanda R., Chow L.Q.M., Dees E.C., Berger R., Gupta S., Geva R., Pusztai L., Pathiraja K., Aktan G., Cheng J.D. (2016). Pembrolizumab in Patients with Advanced Triple-Negative Breast Cancer: Phase Ib KEYNOTE-012 Study. J. Clin. Oncol..

[14] Adams S., Loi S., Toppmeyer D., Cescon D.W., De Laurentiis M., Nanda R., Winer E.P., Mukai H., Tamura K., Armstrong A. (2017). Phase 2 study of pembrolizumab as first-line therapy for PD-L1–positive metastatic triple-negative breast cancer (mTNBC): Preliminary data from KEYNOTE-086 cohort B. J. Clin. Oncol..

[15] Roche Group Media Relations (2020). Roche Provides Update on Phase III Study of Tecentriq in Combination with Paclitaxel for People with Metastatic Triple-Negative Breast Cancer.

[16] Adams S., Diamond J., Hamilton E., Pohlmann P., Tolaney S., Molinero L., Zou W., Liu B., Waterkamp D., Funke R. (2016). Abstract P2-11-06: Safety and clinical activity of atezolizumab (anti-PDL1) in combination with nab-paclitaxel in patients with metastatic triple-negative breast cancer. Cancer Res..

[17] Schmid P., Adams S., Rugo H.S., Schneeweiss A., Barrios C.H., Iwata H., Diéras V., Hegg R., Im S.-A., Shaw Wright G. (2018). Atezolizumab and Nab-Paclitaxel in Advanced Triple-Negative Breast Cancer. N. Engl. J. Med..

[18] Cortes J., Cescon D.W., Rugo H.S., Nowecki Z., Im S.A., Yusof M.M., de las Heras Begona B. (2020). Pembrolizumab plus chemotherapy versus placebo plus chemotherapy for previously untreated locally recurrent inoperable or metastatic triple-negative breast cancer (KEYNOTE-355): A randomised, placebo-controlled, double-blind, phase 3 clinical trial. Clin. Trial.

[19] Cortés J., Cescon D., Rugo H., Im S.-A., Yusof M.M., Gallardo C., Lipatov O., Barrios C., Perez-Garcia J., Iwata H. (2021). LBA16 KEYNOTE-355: Final results from a randomized, double-blind phase III study of first-line pembrolizumab + chemotherapy vs placebo + chemotherapy for metastatic TNBC. Ann. Oncol..

[20] Rugo H.S., Delord J.-P., Im S.-A., Ott P.A., Piha-Paul S., Bedard P., Sachdev J., Le Tourneau C., Van Brummelen E.M., Varga A. (2018). Safety and Antitumor Activity of Pembrolizumab in Patients with Estrogen Receptor–Positive/Human Epidermal Growth Factor Receptor 2–Negative Advanced Breast Cancer. Clin. Cancer Res..

[21] Dirix L.Y., Takacs I., Jerusalem G., Nikolinakos P., Arkenau H.T., Forero-Torres A., Boccia R., Lippman M.E., Somer R., Smakal M. (2018). Avelumab, an anti-PD-L1 antibody, in patients with locally advanced or metastatic breast cancer: A phase 1b JAVELIN Solid Tumor study. Breast Cancer Res. Treat..

[22] Tolaney S.M., Barroso-Sousa R., Keenan T., Li T., Trippa L., Vaz-Luis I., Wulf G., Spring L., Sinclair N.F., Andrews C. (2020). Effect of eribulin with or without pembrolizumab on progression-free survival for patients with hormone receptor–positive, ERBB2-negative metastatic breast cancer: A randomized clinical trial. JAMA Oncol..

[23] Loi S., Giobbie-Hurder A., Gombos A., Bachelot T., Hui R., Curigliano G., Campone M., Biganzoli L., Bonnefoi H., Jerusalem G. (2019). Pembrolizumab plus trastuzumab in trastuzumab-resistant, advanced, HER2-positive breast cancer (PANACEA): A single-arm, multicentre, phase 1b–2 trial. Lancet Oncol..

[24] Emens L., Esteva F., Beresford M., Saura C., De Laurentiis M., Kim S.-B., Im S.-A., Wang Y., Mani A., Shah J. (2019). Overall survival (OS) in KATE2, a phase II study of programmed death ligand 1 (PD-L1) inhibitor atezolizumab (atezo)+trastuzumab emtansine (T-DM1) vs placebo (pbo)+T-DM1 in previously treated HER2+ advanced breast cancer (BC). Ann. Oncol..

[25] Xing D., Siva S., Hanna G. (2019). The abscopal effect of stereotactic radiotherapy and immunotherapy: Fool’s gold or El dorado?. Clin. Oncol..

[26] Siva S., MacManus M., Martin R.F., Martin O.A. (2015). Abscopal effects of radiation therapy: A clinical review for the radiobiologist. Cancer Lett..

[27] Reits E.A., Hodge J.W., Herberts C.A., Groothuis T.A., Chakraborty M., Wansley E.K., Camphausen K., Luiten R.M., De Ru A.H., Neijssen J. (2006). Radiation modulates the peptide repertoire, enhances MHC class I expression, and induces successful antitumor immunotherapy. J. Exp. Med..

[28] Obeid M., Tesniere A., Ghiringhelli F., Fimia G.M., Apetoh L., Perfettini J.-L., Castedo M., Mignot G., Panaretakis T., Casares N. (2007). Calreticulin exposure dictates the immunogenicity of cancer cell death. Nat. Med..

[29] Yoshimoto Y., Oike T., Okonogi N., Suzuki Y., Ando K., Sato H., Noda S.-E., Isono M., Mimura K., Kono K. (2015). Carbon-ion beams induce production of an immune mediator protein, high mobility group box 1, at levels comparable with X-ray irradiation. J. Radiat. Res..

[30] Kondo T., Kobayashi J., Saitoh T., Maruyama K., Ishii K.J., Barber G.N., Komatsu K., Akira S., Kawai T. (2013). DNA damage sensor MRE11 recognizes cytosolic double-stranded DNA and induces type i interferon by regulating STING trafficking. Proc. Natl. Acad. Sci. USA.

[31] Vanpouille-Box C., Alard A., Aryankalayil M.J., Sarfraz Y., Diamond J.M., Schneider R.J., Inghirami G., Coleman C.N., Formenti S.C., DeMaria S. (2017). DNA exonuclease Trex1 regulates radiotherapy-induced tumour immunogenicity. Nat. Commun..

[32] Lugade A.A., Sorensen E., Gerber S.A., Moran J.P., Frelinger J.G., Lord E.M. (2008). Radiation-Induced IFN-γ Production within the Tumor Microenvironment Influences Antitumor Immunity. J. Immunol..

[33] Matzinger P. (1994). Tolerance, danger, and the extended family. Annu. Rev. Immunol..

[34] Matzinger P. (2002). The Danger Model: A Renewed Sense of Self. Science.

[35] Kachikwu E.L., Iwamoto K.S., Liao Y.-P., DeMarco J.J., Agazaryan N., Economou J.S., McBride W.H., Schaue D. (2011). Radiation Enhances Regulatory T Cell Representation. Int. J. Radiat. Oncol. Biol. Phys..

[36] Tanaka H., Shinto O., Yashiro M., Yamazoe S., Iwauchi T., Muguruma K., Kubo N., Ohira M., Hirakawa K. (2010). Transforming growth factor β signaling inhibitor, SB-431542, induces maturation of dendritic cells and enhances anti-tumor activity. Oncol. Rep..

[37] Tsai C.-S., Chen F.-H., Wang C.-C., Huang H.-L., Jung S.-M., Wu C.-J., Lee C.-C., McBride W.H., Chiang C.-S., Hong J.-H. (2007). Macrophages from Irradiated Tumors Express Higher Levels of iNOS, Arginase-I and COX-2, and Promote Tumor Growth. Int. J. Radiat. Oncol. Biol. Phys..

[38] Xu J., Escamilla J., Mok S., David J.R., Priceman S.J., West B., Bollag G., McBride W.H., Wu L. (2013). CSF1R Signaling Blockade Stanches Tumor-Infiltrating Myeloid Cells and Improves the Efficacy of Radiotherapy in Prostate Cancer. Cancer Res..

[39] Shevtsov M., Sato H., Multhoff G., Shibata A. (2019). Novel Approaches to Improve the Efficacy of Immuno-Radiotherapy. Front. Oncol..

[40] Deng L., Liang H., Burnette B., Beckett M., Darga T., Weichselbaum R.R., Fu Y.-X. (2014). Irradiation and anti–PD-L1 treatment synergistically promote antitumor immunity in mice. J. Clin. Investig..

[41] Schaue D., Ratikan J.A., Iwamoto K.S., McBride W.H. (2012). Maximizing Tumor Immunity with Fractionated Radiation. Int. J. Radiat. Oncol. Biol. Phys..

[42] Finkelstein S.E., Timmerman R., McBride W.H., Schaue D., Hoffe S.E., Mantz C.A., Wilson G. (2011). The Confluence of Stereotactic Ablative Radiotherapy and Tumor Immunology. Clin. Dev. Immunol..

[43] Harris T.J., Hipkiss E.L., Borzillary S., Wada S., Grosso J.F., Yen H.-R., Getnet D., Bruno T.C., Goldberg M.V., Pardoll D.M. (2008). Radiotherapy augments the immune response to prostate cancer in a time-dependent manner. Prostate.

[44] Reck M., Bondarenko I., Luft A., Serwatowski P., Barlesi F., Chacko R., Sebastian M., Lu H., Cuillerot J.-M., Lynch T.J. (2013). Ipilimumab in combination with paclitaxel and carboplatin as first-line therapy in extensive-disease-small-cell lung cancer: Results from a randomized, double-blind, multicenter phase 2 trial. Ann. Oncol..

[45] Lynch T.J., Bondarenko I., Luft A., Serwatowski P., Barlesi F., Chacko R., Sebastian M., Neal J., Lu H., Cuillerot J.-M. (2012). Ipilimumab in Combination with Paclitaxel and Carboplatin as First-Line Treatment in Stage IIIB/IV Non–Small-Cell Lung Cancer: Results from a Randomized, Double-Blind, Multicenter Phase II Study. J. Clin. Oncol..

[46] Young K., Cottam B., Baird J., Gough M., Crittenden M. (2014). Ideal Timing of Immunotherapy with Radiation in Murine Tumor Models. Int. J. Radiat. Oncol. Biol. Phys..

[47] Qian J.M., Yu J., Kluger H.M., Chiang V.L.S. (2016). Timing and type of immune checkpoint therapy affect the early radiographic response of melanoma brain metastases to stereotactic radiosurgery. Cancer.

[48] Samstein R., Rimner A., Barker C., Yamada Y. (2017). Combined Immune Checkpoint Blockade and Radiation Therapy: Timing and Dose Fractionation Associated with Greatest Survival Duration Among Over 750 Treated Patients. Int. J. Radiat. Oncol. Biol. Phys..

[49] Demaria S., Ng B., Devitt M.L., Babb J., Kawashima N., Liebes L., Formenti S.C. (2004). Ionizing radiation inhibition of distant untreated tumors (abscopal effect) is immune mediated. Int. J. Radiat. Oncol. Biol. Phys..

[50] Siva S., Callahan J., MacManus M.P., Martin O., Hicks R.J., Ball D.L. (2013). Asbcopal effects after conventional and stereotactic lung irradiation of non-small cell lung cancer. J. Thorac. Oncol..

[51] Formenti S.C., Demaria S. (2009). Systemic effects of local radiotherapy. Lancet Oncol..

[52] Park H.J., Griffin R.J., Hui S., Levitt S.H., Song C.W. (2012). Radiation-induced vascular damage in tumors: Implications of vascular damage in ablative hypofractionated radio-therapy (SBRT and SRS). Radiat. Res..

[53] Fuks Z., Kolesnick R. (2005). Engaging the vascular component of the tumor response. Cancer Cell.

[54] Lee Y., Auh S.L., Wang Y., Burnette B., Meng Y., Beckett M., Sharma R., Chin R., Tu T., Weichselbaum R.R. (2009). Therapeutic effects of ablative radiation on local tumor require CD8^+^ T cells: Changing strategies for cancer treatment. Blood.

[55] Lugade A.A., Moran J.P., Gerber S.A., Rose R.C., Frelinger J.G., Lord E.M. (2005). Local Radiation Therapy of B16 Melanoma Tumors Increases the Generation of Tumor Antigen-Specific Effector Cells That Traffic to the Tumor. J. Immunol..

[56] Dewan M.Z., Galloway A.E., Kawashima N., Dewyngaert J.K., Babb J.S., Formenti S.C., Demaria S. (2009). Fractionated but Not Single-Dose Radiotherapy Induces an Immune-Mediated Abscopal Effect when Combined with Anti–CTLA-4 Antibody. Clin. Cancer Res..

[57] Verbrugge I., Hagekyriakou J., Sharp L.L., Galli M., West A., McLaughlin N.M., Duret H., Yagita H., Johnstone R.W., Smyth M.J. (2012). Radiotherapy Increases the Permissiveness of Established Mammary Tumors to Rejection by Immunomodulatory Antibodies. Cancer Res..

[58] Dushyanthen S., Beavis P., Savas P., Teo Z.L., Zhou C., Mansour M., Darcy P.K., Loi S. (2015). Relevance of tumor-infiltrating lymphocytes in breast cancer. BMC Med..

[59] Spellman A., Tang S.-C. (2016). Immunotherapy for breast cancer: Past, present, and future. Cancer Metastasis Rev..

[60] McBride S.M., Sherman E.J., Tsai C.J., Baxi S.S., Aghalar J., Eng J., Zhi W.I., McFarland D.C., Michel L.S., Spielsinger D. (2018). A phase II randomized trial of nivolumab with stereotactic body radiotherapy (SBRT) versus nivolumab alone in metastatic (M1) head and neck squamous cell carcinoma (HNSCC). J. Clin. Oncol..

[61] Theelen W.S., Peulen H.M., Lalezari F., van der Noort V., De Vries J.F., Aerts J.G., Dumoulin D.W., Bahce I., Niemeijer A.L.N., De Langen A.J. (2019). Effect of pembrolizumab after stereotactic body radiotherapy vs pembrolizumab alone on tumor response in patients with advanced non–small cell lung cancer: Results of the PEMBRO-RT phase 2 randomized clinical trial. JAMA Oncol..

[62] Welsh J.W., Chen D., Baas P., Chang J.Y., Verma V., Comeaux N., Skoulidis F., Lin S.H., Heymach J., Theelen W. (2020). Radiotherapy to augment pembrolizumab responses and outcomes in metastatic non-small cell lung cancer: Pooled analysis of two randomized trials. J. Clin. Oncol..

[63] Savas P., Teo Z.L., Lefevre C., Flensburg C., Caramia F., Alsop K., Mansour M., Francis P., Thorne H., Silva M.J. (2016). The Subclonal Architecture of Metastatic Breast Cancer: Results from a Prospective Community-Based Rapid Autopsy Program “CASCADE”. PLOS Med..

[64] Tang C., Welsh J.W., De Groot P., Massarelli E., Chang J.Y., Hess K.R., Basu S., Curran M., Cabanillas M.E., Subbiah V. (2017). Ipilimumab with Stereotactic Ablative Radiation Therapy: Phase I Results and Immunologic Correlates from Peripheral T Cells. Clin. Cancer Res..

[65] David S., Tan J., Savas P., Bressel M., Kelly D., Foroudi F., Loi S., Siva S. (2020). Stereotactic ablative body radiotherapy (SABR) for bone only oligometastatic breast cancer: A prospective clinical trial. Breast.

[66] Trovo M., Furlan C., Polesel J., Fiorica F., Arcangeli S., Giaj-Levra N., Alongi F., Del Conte A., Militello L., Muraro E. (2018). Radical radiation therapy for oligometastatic breast cancer: Results of a prospective phase II trial. Radiother. Oncol..

[67] Milano M.T., Zhang H., Metcalfe S.K., Muhs A.G., Okunieff P. (2008). Oligometastatic breast cancer treated with curative-intent stereotactic body radiation therapy. Breast Cancer Res. Treat..

[68] Palma D.A., Haasbeek C.J.A., Rodrigues G.B., Dahele M., Lock M., Yaremko B., Olson R., Liu M., Panarotto J., Griffioen G.H. (2012). Stereotactic ablative radiotherapy for comprehensive treatment of oligometastatic tumors (SABR-COMET): Study protocol for a randomized phase II trial. BMC Cancer.

[69] Gustave Roussy Cancer Campus (2014). Trial of Superiority of Stereotactic Body Radiation Therapy in Patients with Breast Cancer (STE-REO-SEIN). https://clinicaltrials.gov/ct2/show/NCT02089100.

[70] NRG Oncology (2015). Standard of Care Therapy with or Without Stereotactic Radiosurgery and/or Surgery in Treating Patients with Limited Metastatic Breast Cancer. https://clinicaltrials.gov/ct2/show/record/NCT02364557.

[71] Krug D., Vonthein R., Illen A., Olbrich D., Barkhausen J., Richter J., Klapper W., Schmalz C., Rody A., Maass N. (2021). Metastases-directed Radiotherapy in Addition to Standard Systemic Therapy in Patients with Oligometastatic Breast Cancer: Study protocol for a randomized controlled multi-national and multi-center clinical trial (OLIGOMA). Clin. Transl. Radiat. Oncol..

[72] Thureau S., Marchesi V., Vieillard M.-H., Perrier L., Lisbona A., Leheurteur M., Tredaniel J., Culine S., Dubray B., Bonnet N. (2021). Efficacy of extracranial stereotactic body radiation therapy (SBRT) added to standard treatment in patients with solid tumors (breast, prostate and non-small cell lung cancer) with up to 3 bone-only metastases: Study protocol for a randomised phase III trial (STEREO-OS). BMC Cancer.

[73] Khoo V., Hawkins M., Ahmed M., Kirby A., Van As N., McDonald F., Franks K., Syndikus I., Jain S., Tree A. (2018). A randomised trial of conventional care versus radioablation (stereotactic body radiotherapy) for extracranial oli-gometastases. Clin. Oncol..

[74] Palma D.A., Olson R., Harrow S., Correa R.J.M., Schneiders F., Haasbeek C.J.A., Rodrigues G.B., Lock M., Yaremko B.P., Bauman G.S. (2019). Stereotactic ablative radiotherapy for the comprehensive treatment of 4–10 oligometastatic tumors (SABR-COMET-10): Study protocol for a randomized phase III trial. BMC Cancer.

[75] Tarantino P., Marra A., Gandini S., Minotti M., Pricolo P., Signorelli G., Criscitiello C., Locatelli M., Belli C., Bellomi M. (2020). Association between baseline tumour burden and outcome in patients with cancer treated with next-generation immunoncology agents. Eur. J. Cancer.

[76] Gutiontov S.I., Pitroda S.P., Chmura S.J., Arina A., Weichselbaum R.R. (2020). Cytoreduction and the Optimization of Immune Checkpoint Inhibition with Radiation Therapy. Int. J. Radiat. Oncol. Biol. Phys..

[77] Siva S., Bressel M., Wood S.T., Shaw M.G., Loi S., Sandhu S.K., Tran B., Azad A.A., Lewin J.H., Cuff K.E. (2021). Stereotactic Radiotherapy and Short-course Pembrolizumab for Oligometastatic Renal Cell Carcinoma—The RAPPORT Trial. Eur. Urol..

[78] McDermott D.F., Lee J.-L., Bjarnason G.A., Larkin J.M.G., Gafanov R.A., Kochenderfer M.D., Jensen N.V., Donskov F., Malik J., Poprach A. (2021). Open-Label, Single-Arm Phase II Study of Pembrolizumab Monotherapy as First-Line Therapy in Patients with Advanced Clear Cell Renal Cell Carcinoma. J. Clin. Oncol. Off. J. Am. Soc. Clin. Oncol..

[79] Faivre-Finn C., Vicente D., Kurata T., Planchard D., Paz-Ares L., Vansteenkiste J.F., Spigel D.R., Garassino M.C., Reck M., Senan S. (2021). Four-Year Survival with Durvalumab After Chemoradiotherapy in Stage III NSCLC—An Update from the PACIFIC Trial. J. Thorac. Oncol..

[80] Formenti S.C., Lee P., Adams S., Goldberg J.D., Li X., Xie M.W., Ratikan J.A., Felix C., Hwang L., Faull K.F. (2018). Focal irradiation and systemic TGFβ blockade in metastatic breast cancer. Clin. Cancer Res..

[81] Ho A.Y., Barker C.A., Arnold B.B., Powell S.N., Hu Z.I., Gucalp A., Lebron-Zapata L., Wen H.Y., Kallman C., D’Agnolo A. (2020). A phase 2 clinical trial assessing the efficacy and safety of pembrolizumab and radiotherapy in patients with metastatic triple-negative breast cancer. Cancer.

[82] Voorwerk L., Slagter M., Horlings H.M., Sikorska K., Van De Vijver K.K., De Maaker M., Nederlof I., Kluin R.J.C., Warren S., Ong S. (2019). Immune induction strategies in metastatic triple-negative breast cancer to enhance the sensitivity to PD-1 blockade: The TONIC trial. Nat. Med..

[83] Kritz-Silverstein D., Von Mühlen D., Barrett-Connor E., Bressel M.A.B. (2003). Isoflavones and cognitive function in older women: The SOy and Postmenopausal Health in Aging (SOPHIA) Study. Menopause.

[84] Harini D., de Silva P.S., Petrone P., Neeson M., Tantalo D., Siva S., Bressel M., David S., Neeson P.J., Loi S. Identification of peripheral blood biomarkers that predict response to the combination of radiotherapy and anti-PD1 checkpoint blockade in metastatic breast cancer. Proceedings of the ASI 48th Annual Scientific Meeting of the Australian and New Zealand Society for Immunology.

